# Impact of Local Composition on the Emission Spectra of InGaN Quantum-Dot LEDs

**DOI:** 10.3390/nano13081367

**Published:** 2023-04-14

**Authors:** Daniele Barettin, Alexei V. Sakharov, Andrey F. Tsatsulnikov, Andrey E. Nikolaev, Alessandro Pecchia, Matthias Auf der Maur, Sergey Yu. Karpov, Nikolay Cherkashin

**Affiliations:** 1 Department of Electronic Engineering, Università Niccoló Cusano, 00133 Rome, Italy; 2 Ioffe Physico-Technical Institute RAS, 26 Polytekhnicheskaya str., 194021 St. Petersburg, Russia; val.beam@mail.ioffe.ru (A.V.S.); andrew@beam.ioffe.ru (A.F.T.); Aen@mail.ioffe.ru (A.E.N.); 3 CNR-ISMN, Via Salaria Km. 29.300, Monterotondo, 00017 Rome, Italy; alessandro.pecchia@cnr.it; 4 Department of Electronic Engineering, University of Rome Tor Vergata, Via del Politecnico 1, 00133 Rome, Italy; 5 Soft-Impact, Ltd., P.O. Box 83, 27 Engels ave., 194156 St. Petersburg, Russia; 6 CEMES-CNRS and Université de Toulouse, 29 rue Jeanne Marvig, BP 94347, F-31055 Toulouse, CEDEX 4, France

**Keywords:** quantum dots, $\vec{k}\cdot \vec{p}$, empirical tight-binding, modeling

## Abstract

A possible solution for the realization of high-efficiency visible light-emitting diodes (LEDs) exploits InGaN-quantum-dot-based active regions. However, the role of local composition fluctuations inside the quantum dots and their effect of the device characteristics have not yet been examined in sufficient detail. Here, we present numerical simulations of a quantum-dot structure restored from an experimental high-resolution transmission electron microscopy image. A single InGaN island with the size of ten nanometers and nonuniform indium content distribution is analyzed. A number of two- and three-dimensional models of the quantum dot are derived from the experimental image by a special numerical algorithm, which enables electromechanical, continuum $\vec{k}\cdot \vec{p}$, and empirical tight-binding calculations, including emission spectra prediction. Effectiveness of continuous and atomistic approaches are compared, and the impact of InGaN composition fluctuations on the ground-state electron and hole wave functions and quantum dot emission spectrum is analyzed in detail. Finally, comparison of the predicted spectrum with the experimental one is performed to assess the applicability of various simulation approaches.

## 1. Introduction

To form active regions of visible LEDs for solid-state lighting, a large number of solutions has been suggested, including [1,2]. Among them, indium gallium nitride (InGaN) alloys have become of special importance due to demonstrated high emission efficiency, compatibility with conventional epitaxial techniques, and covering the major part of the visible spectral range [3,4,5,6,7]. First of all, this was due to a high internal quantum efficiency (IQE), attributed to the lateral electron and hole localization originating from fluctuation of either composition [8] or width of InGaN quantum wells (QWs) embedded in the active region [9]. Another evident reason was the possibility to cover the whole visible spectrum range by engineering the In content in the QWs [10,11].

However, this material system still faces some problems. First, the efficiency decreases in the green and yellow/amber spectral range, as compared to the blue one (the phenomenon known as “green gap”), which is still under investigation [12,13,14]. Second, the relevant lattice constant mismatch (≈10%) between InN and GaN makes a pseudomorphic growth of QWs with high In content problematic due to strain relaxation [15], enhanced indium segregation [16], enhanced fluctuations of QW widths, and InGaN composition [17,18], since these factors favor likely generation of dislocations and other defects in the active region, reducing IQE of the LEDs [19]. Finally, InGaN/GaN wurtzite heterostructures, conventionally grown along the [0001] direction, exhibit strong built-in electric fields inside the QWs, producing a relevant quantum-confined Stark effect (QCSE): a tilt of the conduction and valence bands resulted in a reduced overlap of electron and hole wave functions and, eventually, in a decrease of the efficiency of radiative recombination in the LED [20,21,22,23].

A possible workaround of the above problems is the fabrication of quantum-dot-based active regions, which can be grown with a higher In content compared to QWs, due to reduction of the accumulated elastic energy via defect-free stress relaxation at the edges of the dots. This solution would also make it possible to extend the spectral range of InGaN-based LEDs, being limited by the bandgaps of binary compounds InN and GaN [10,11]. The quantum dots (QDs), i.e., the InGaN islands embedded in the GaN matrix, can be manufactured, in particular, by using a technologically controlled growth interruption technique once a 2D InGaN layer has been formed [24].

Several models have been suggested to study the electronic structure of wurtzite In${}_{x}$Ga${}_{1-x}$ N QDs including the effect of electromechanical field. The models use both the continuous approach, starting from the paper by Winkelnkemper et al. [25], who extended the model of Chuang and Chang [26], and atomistic simulations, which are supposed to include the real symmetry of the structure, such as a tight-binding model accounting for electromechanical fields in In${}_{x}$Ga${}_{1-x}$ N/GaN QDs [27], GaN/AlN QDs [28], and pure InN/GaN QDs [29].

Some papers from our group and coauthors have recently shown that accounts of the realistic strain distribution [30] and quantum confinement [31,32] in a nanostructure are fundamental for the accurate theoretical predictions and interpretation of experimental results. In this context, we have already presented a realistic model of an InGaN/GaN LED structure given by a thick n-GaN contact layer, a prelayer made of a 12-period short-period InGaN/GaN superlattice (SPSL), undoped GaN spacer, active region (AR) including double-stacked QD layers, p-AlGaN electron-blocking layer (EBL), and p-GaN contact layer. The model was quite suitable for studying the impact of the lateral and vertical QD coupling via electromechanical field [33] and possible extension of the wavelength emission range [34].

The present paper is aimed at analyzing the influence of InGaN compositional inhomogeneity in the QD on its optical properties. In fact, this aspect has been studied in quantum wells and more specifically in LED-based InGaN quantum-wells [35,36,37,38]. However, we are not yet aware of any publications that would thoroughly investigate this phenomenon in QDs, where it could significantly influence the quantum confinement of carriers and spatial localization of their wave functions. For this purpose, we applied both continuum models based on the $\vec{k}\cdot \vec{p}$ approach [39,40] and the atomistic model based on empirical tight-binding calculations [41,42] within a multiscale approach [43].

The paper is organized in the following way. In Section 2, we describe the procedure for generation of the two- and three-dimensional QD structures to be used in simulations. The models applied are considered in detail in Section 3. The results of simulations are discussed in Section 4, whereas our conclusions complete the paper.

## 2. Structures for Quantum Dots

The active region of the structure under study contains a double layer of InGaN islands formed by the growth-interruption approach. It is sandwiched between a 12 × {1 nm GaN/1 nm InGaN} period superlattice and undoped GaN spacer from the bottom side and a p-AlGaN electron blocking layer covered by a p-GaN contact layer from the top surface side.

Two- and three-dimensional reconstruction of the simulated QD structure was based on a special algorithm operating with an experimental image of the out-of-plane strain derived by the geometric phase analysis (GPA) of the high-resolution transmission electron microscopy (HRTEM) image of a real QD structure [44]. The sample used for this purpose, as was already mentioned, contained several InGaN/GaN superlattices and large InGaN QD islands with the sizes of tens nanometers and nonuniform InGaN composition distribution.

For the present study, we sampled a single QD from the active region using the GwyddyonTM software [45], and then we reconstructed the two- and three-dimensional structures for simulation. The latter one was obtained within a rotation-invariance approximation, i.e., by rotation of two-dimensional structure around the hexagonal axis of the crystal. We suppose that such a three-dimensional structure was not far from the experimental one exhibiting a hexagonal or dodecagonal symmetry. The reconstruction method was described in detail in Ref. [31]. A finite element method (FEM) was subsequently applied to the reconstructed structures in order to discretize the electromechanical, electronic, and optical models. The entire experimental procedure for deriving the experimental out-of-plane strain distribution by the GPA of the QD HRTEM image and then extracting the reconstructed structures by a special algorithm was described and discussed in detail in Ref. [33].

**Figure 1:**
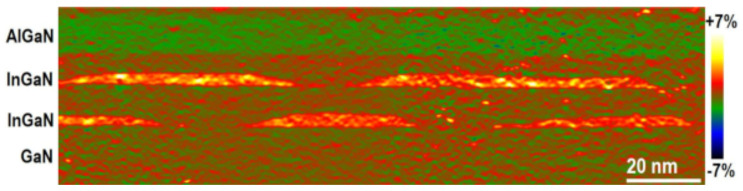
The two-dimensional image of the out-of-plane strain obtained by geometric phase analysis of the HRTEM image used to derive the three-dimensional structure.

The two-dimensional image of the out-of-plane strain of the experimental sample, from which two- and three-dimensional simulations structures were obtained, is shown in Figure 1. This sample was also used in our previous publications on the electromechanical coupling and wavelength emission range [33,34].

## 3. Models

Calculations of the electromechanical fields were made according to the theories described in detail in Ref. [46]. In the continuum models, the total free energy density is used to derive the governing equations for the electromechanical fields of the crystal based on Newton’s second law and Maxwell–Poisson equation, i.e., a set of four coupled equations for the electromechanical fields. The results were then used in the framework of the $\vec{k}\cdot \vec{p}$ model via deformation potentials [47].

In the atomistic approach, the valence-force-field (VFF) model provided the equilibrium atomic positions by minimizing the QD elastic energy, taking into account bond stretching and bond bending forces. The strain effects were then included in the empirical tight-binding (ETB) model with the Harrison-type scaling laws for fitted exponents [48].

The piezoelectric field was included directly in the continuum model, whereas it was calculated by a semicoupled model from the VFF results, which did not account for long-ranging Coulomb effects. Nevertheless, the atomistic VFF method and continuous model showed an excellent agreement with each other in the case of the strain field calculations for relatively large QDs [46,49].

Since our study is largely focused on investigating the impact of InGaN composition fluctuations in QDs on their emission spectra, we used single-particle models for band structure, neglecting Coulomb coupling and possible decoherence effects due to phonons [50]. In the continuum approach, the band structure calculations were based on the 8-band $\vec{k}\cdot \vec{p}$ model [39,40], which described electron, heavy-hole, light-hole, and spin-orbit split-off bands around the Γ point of the Brillouin zone, all other bands being considered as remote ones. The Hamiltonian is derived by Foreman’s application of Burt’s envelope function for heterostructures [51,52]. The necessary band structure parameters were borrowed from Ref. [53].

Within this approach, the wave function of a state *n* with energy $E_{n}$ was represented as a linear combination of eight Bloch magnitudes weighted by the respective envelope functions,

(1) $$\begin{array}{c} \psi_{n}=\sum_{i=1}^{8}\phi_{i}u_{i}, \end{array}$$

where $\phi_{i}$ are the envelope function and $u_{i}$ are the Bloch magnitudes [54]. We solved the $\vec{k}\cdot \vec{p}$ equations using the finite element method (FEM), accounting for the electromechanical field. Following the continuum approach, for the computation of optical transitions since the dipole matrix elements ${\vec{\mu }}_{nm}$ are ill defined in crystals involving extended Bloch states [52], these were calculated from the momentum matrix element

(2) $$\begin{array}{cc} {\vec{p}}_{nm}\; & \equiv \langle \psi_{n}|\vec{p}\;|\psi_{m}\rangle \\ \; & =\sum_{i,j=1}^{8}\left(\langle \phi_{i}^{\left(n\right)}|\vec{p}\;|\phi_{j}^{\left(m\right)}\rangle \delta_{ij}+\langle \phi_{i}^{\left(n\right)}|\phi_{j}^{\left(m\right)}\rangle \langle u_{i}|\vec{p}\;|u_{j}\rangle \right)\\ & \equiv {\vec{p}}_{nm}^{\;(\phi )}+{\vec{p}}_{nm}^{\;(u)}, \end{array}$$

where ${\vec{p}}^{\;(\phi )}$ and ${\vec{p}}^{\;(u)}$ are the envelope and the Bloch constituents of the momentum matrix element, respectively. The envelope functions were usually slowly varying at the scale of the primitive cell, so it was possible to neglect them [55,56]. The oscillator strengths were finally given by [57]:

(3) $$\begin{array}{c} {\vec{P}}_{nm}=\frac{2\pi e^{2}\hbar^{2}}{\epsilon_{0}m_{0}^{2}V}{\left|\hat{e}\cdot {\vec{p}}_{nm}\right|}^{2}, \end{array}$$

where $\hat{e}$ is the direction unit vector of the electric field, *V* is the volume of the structure, *e* is the electron charge, $m_{0}$ is the free electron mass, and $\epsilon_{0}$ is the vacuum permittivity.

The ETB method is usually based on precomputed parameters for the two-center interactions between the atomic orbitals [41]. Our model uses a parameterization based on a sp${}^{3}$s${}^{*}$d${}^{5}$ local basis, as established in Ref. [48], where the parameters are fitted to experimental data and bulk dispersions obtained by density functional theory. The spin-orbit coupling is included within each atomic center [58].

Considering ETB optical transitions, we followed the work of Ref. [59], where momentum matrix elements are evaluated as

(4) $$\begin{array}{c} {\vec{p}}_{nm}\left(k\right)=\frac{m_{0}}{\hbar }\sum_{b\alpha b^{\prime }\alpha^{\prime }}C_{nk}^{*}\left(b,\alpha \right)C_{mk}\left(b^{\prime },\alpha^{\prime }\right)\nabla_{k}H_{b\alpha ,b^{\prime }\alpha^{\prime }\left(k\right)}, \end{array}$$

where n,m are eigenstate indices while b,α denotes an atomic orbital α centered at ${\vec{R}}_{b}$, and in the momentum representation ${\vec{\nabla }}_{\vec{k}}H_{b\alpha ,b^{\prime }\alpha^{\prime }\left(k=0\right)}=\left({\vec{R}}_{b}-{\vec{R}}_{b^{\prime }}\right)$. The Equation (4) neglected intra-atomic terms and spin-orbit corrections. Generally, it is still controversial as to how optical transitions should be treated in the ETB approximation. Therefore, we refer one to the existing literature for a more detailed discussion [60,61,62,63]. All models were implemented and solved by using the TiberCAD simulator [64,65].

## 4. Results

A preliminary comparison of the experimentally measured strain field $\varepsilon_{zz}$ relative to the GaN substrate with the predictions of the continuum model allowed us to obtain the actual average In content of the dots, which was estimated to be approximately 19%. Having established this fact, the first step of our study was to qualitatively analyze the impact of local In content on the optical properties of an InGaN QD, comparing four different two-dimensional models.

**Figure 2:**
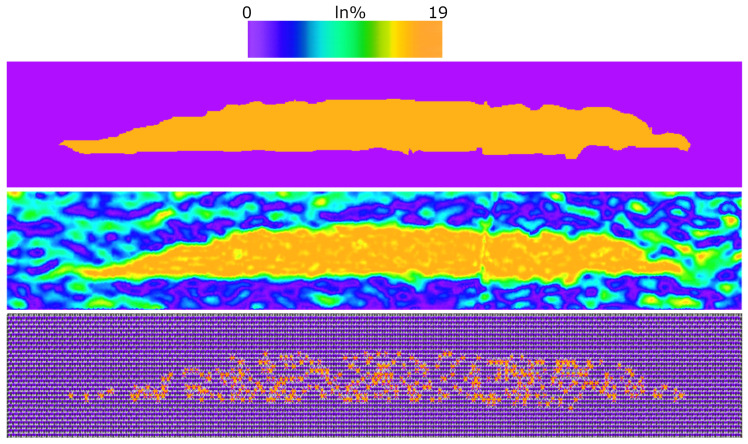
Two-dimensional distributions of the In content in the QD. (**Top**): the structure with a uniform In distribution of (19%). (**Center**): the structure derived by an algorithm accounting for the experimental local In content. (**Bottom**): the structure with an atomistic homogeneous random In distribution in InGaN QD with the mean content of 19% (bigger orange spots correspond to In atoms).

The first two cases considered are (i) QD with a uniform In content of 19% in the whole structure and (ii) QD with a realistic nonuniform In content distribution given by the data, obtained with an ad hoc algorithm reconstructing the actual local alloy composition. The corresponding In composition profiles are shown in Figure 2.

The simulations for these two configurations were performed with a continuum model for the electromechanical field [46] and 8-band $\vec{k}\cdot \vec{p}$ model for optoelectronic properties [40]. The strain distribution inside the QD is closely related to the local In content. In the case of a uniform In distribution in the QD, the strain field is also highly uniform and rapidly tends to zero outside the QD. In the case of the real (experimental) In content distribution, the strain fluctuates inside the QD and does not disappear outside of the dot, where the In content is still nonzero [33].

For the last two cases, we considered results obtained by the ETB model with sp3d5s* parameterization [41,42] and atomistic strain field calculated by the VFF method [66], which allow for modeling, in detail, the real indium distributions on the atomic scale. Here, the local strain was also included in the simulations in order to obtain more accurate electronic band structures [37,67]. We implemented two different atomistic approximations in order to include the effect of local InGaN composition fluctuations: the virtual crystal approximation (VCA) and the random alloy (RA) approach. VCA considers an ABC alloy as a fictitious material whose properties are a weighted average of the properties of binary constituents AC and BC, according to Vegard’s law [68]. RA uses the properties averaging over numerous stochastic configurations where the anion A occupies a lattice site with the probability equal to its molar fraction in the alloy. Here, we considered 30 different random configurations within the RA approach. In the bottom of Figure 2, we plot one of the RA configurations, corresponding to the mean 19% In content.

**Figure 3:**
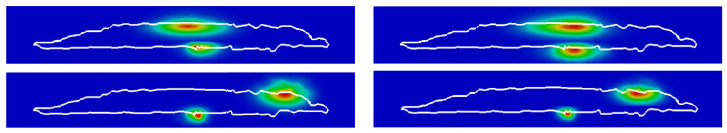
Simulations with two-dimensional models. (**Top**): GS for electrons and holes in the case of $\vec{k}\cdot \vec{p}$ for uniform In content (**left**) and VCA (**right**). (**Bottom**): GS for electrons and holes in the case of $\vec{k}\cdot \vec{p}$ for real In content (**left**) and for one RA configuration (**right**).

Figure 3 presents two-dimensional ground-state (GS) wave functions of electrons (adjacent to the top boundary of QD) and holes (adjacent to the bottom boundary of QD).

Such an adjacency originates from the well-known QCSE in a wurtzite-symmetry structure, since the electric field due to spontaneous polarization and piezoelectric effect shifts the electron and hole wave functions to the opposite sides of the QDs [20,21,22,23,33]. All the panels in the figure demonstrate the localization of the carrier probability densities. However, the localization character is quite different in the cases of (i) the $\vec{k}\cdot \vec{p}$ model with uniform In content distribution or VCA approach and (ii) the $\vec{k}\cdot \vec{p}$ model with real In content distribution or RA approach. In the former case, the electron and hole wave functions are localized in the most wide region of the InGaN QD where the carriers exhibit a weaker quantum confinement. There, the asymmetry of the wave functions and their localization radii are influenced by particular local fluctuations of the QD width. In the latter case, the electron wave function is localized near the edge of the QD, being controlled by local fluctuations of both QD width and composition; the hole localization is nearly the same as in the former case.

**Figure 4:**
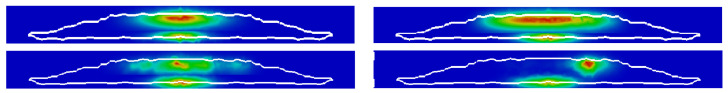
Simulations with three-dimensional models. (**Top**): GS for electrons and holes in the case of $\vec{k}\cdot \vec{p}$ for uniform In content (**left**) and VCA (**right**). (**Bottom**): GS for electrons and holes in the case of one RA configuration in the xz (**left**) and yz (**right**) planes.

To exclude the artifacts that may be an effect of a two-dimensional quantization, we repeated all the simulations using a three-dimensional structure of the QD instead of a two-dimensional one. The corresponding GS wave functions of electrons and holes are shown in Figure 4.

The results obtained do not differ remarkably from those obtained by two-dimensional models. Again, the results coming from the $\vec{k}\cdot \vec{p}$ model with uniform In content distribution and from the VCA approach are similar to each other and differ from the two-dimensional results only by the lateral extensions of the electron and hole wave functions (as previously, all the wave functions were located in the widest part of the QD). The RA approach provides qualitatively different characters of carrier localization, with electrons to be localized aside the central part of the QD. Since we do not have an experimental three-dimensional distribution of the In content available, it was not possible to perform a $\vec{k}\cdot \vec{p}$ calculation for this case.

The above results enable us to make an important conclusions. The trends of electron and hole localization depend substantially on particular InGaN composition distribution in the QD. Therefore, the account of realistic In content distribution is a fundamental aspect, regardless of the model and the choice of a two- or three-dimensional QD structure.

The localization of carriers affects the optical properties of the QD. It is to be expected that the models with a uniform InGaN composition distribution, i.e., continuous ($\vec{k}\cdot \vec{p}$ model and VCA, would present similar overlaps of the electron and hole wave functions and, thus, comparable optical strengths. On the contrary, the results given by the $\vec{k}\cdot \vec{p}$ model with experimental local In content distribution and ETB with random-alloy atomic distribution, which mimics a realistic In content, would show a reduction in the overlaps and, consequentially, in the intensities of the optical strengths.

**Figure 5:**
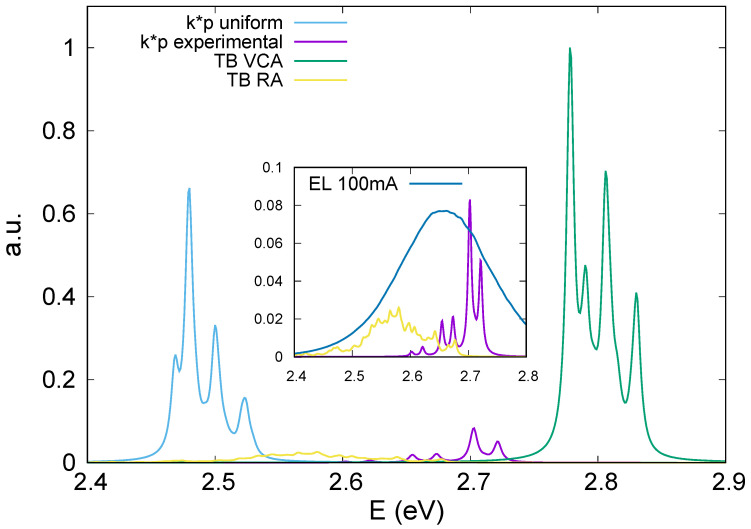
Optical strengths as a function of optical transition energy given by the $\vec{k}\cdot \vec{p}$ models with uniform and real In content distributions and by the ETB approach with VCA and RA composition distributions.

This is exactly what we see in Figure 5, where the optical strengths for all the two-dimensional cases given by the $\vec{k}\cdot \vec{p}$ model with uniform and experimental In content distributions and those obtained by ETB model within VCA and RA approximation are plotted as a function of the optical transition energy. Dependence of the electron and hole states on the local InGaN composition fluctuations and different spatial localization of the carrier wave functions, which are accounted for by the $\vec{k}\cdot \vec{p}$ and ETB models with real In content distribution, results in much lower, approximately by a factor of ten, magnitudes of the optical strengths compared to those assumed to have a uniform distribution. Furthermore, VCA with a linear interpolation of the bandgap considerably overestimates the transition energy [38], whereas the $\vec{k}\cdot \vec{p}$ model with uniform In content underestimates it, as it considers the In content to be systematically greater than the real one, providing an incorrect quantum confinement.

The inset in Figure 5 shows the comparison of our simulations with the experimental room-temperature electroluminescence (EL) spectrum obtained from the studied QD LED structure [33]. The following observations can be made from the comparison. First, one can see that the peak of the experimental EL spectrum is red-shifted by about 60 meV with respect to the principal double-peak of the optical strength obtained with the real In content distribution in the structure and zero temperature. This value is close to the red shift of 65 meV that corresponded to the temperature rise from zero to 300 K and was estimated by the experimental Varshni parameters reported for GaN and InN and bowing parameter for the bandgap of the InGaN alloy. Second, the optical strength spectrum calculated for RA configurations lies well below the above double peak obtained by the $\vec{k}\cdot \vec{p}$ model with the real In content distribution, and likely forms the low-energy wing of the experimental EL spectrum. This may be attributed to the fact that 30 different configurations were accounted for to obtain the optical strength spectra in the RA ETB model, whereas the only InGaN composition distribution averaged over the thickness of the sample studied by TEM was used for realistic $\vec{k}\cdot \vec{p}$ calculations. From such a point of view, the realistic $\vec{k}\cdot \vec{p}$ and RA tight-binding calculations should be regarded as complementary rather then alternative. In particular, the RA calculations may quantitatively explain a wide low-energy wing of the experimental EL spectrum, associating it with InGaN composition fluctuations inherent in RA. In general, both realistic $\vec{k}\cdot \vec{p}$ and RA simulations quite accurately predict the spectral extent of the optical strength. Therefore, account of the local realistic parameters is vital for this kind of simulation, regardless of whether using an atomistic or a continuous method. In this context, a future development of our model will combine the local distribution of a material given by experimental data with the atomic distribution, in order to reduce the statistical fluctuations of the RA model.

## 5. Conclusions

We reported on continuum and atomistic models of optoelectronic properties of a single QD, exploiting two- and three-dimensional QD structures derived from an HRTEM image with nonuniform InGaN composition distribution inside. We compared continuous and atomistic simulations with uniform (mean) and nonuniform (real) In content distributions, analyzing the impact of the local In content on optical properties of the QD.

We found different characters of GS electron and hole localization inside the QD. In the models with a uniform distribution, electrons and holes are localized in the widest regions of the QD, being primarily controlled by the width fluctuations. In the models with nonuniform distribution, fluctuations of the InGaN composition in the QD are likely the main reason for localization of electrons and holes in spatially different regions of the QD.

These different carrier localizations result immediately in a much lower magnitude of the optical transition strengths calculated with accounting of the nonuniform In content distribution compared to the the uniform case. Moreover, the VCA atomistic model is found to overestimate the energies of optical transitions in the QD. In contrast, the $\vec{k}\cdot \vec{p}$ model with a uniform mean In content leads to underestimation of the optical-transition energies. Comparison of the optical strength distributions obtained by both the $\vec{k}\cdot \vec{p}$ model utilizing the real In content distribution and the atomistic ETB model within RA approximation and experimental emission spectrum demonstrated their reasonable agreement. This shows that accounting for the local composition fluctuations in InGaN QDs is the principal and important aspect for this kind of simulation, regardless of the use of either atomistic or continuous models.

## Data Availability

The data presented in the current work are available on request from corresponding authors.

## Abbreviations

The following abbreviations are used in this manuscript:

LED	Light-emitting diode
QWs	Quantum wells
QDs	Quantum dots
AR	Active region
IQE	External quantum efficiency
QCSE	Quantum-confined Stark effect
SPSL	Short-period superlattice
EBL	Electron blocking layer
GPA	Geometric phase analysis
HRTEM	High-resolution transmission electron microscopy
FEM	Finite element model
VFF	Valence force field
ETB	Empirical tight-binding
VCA	Virtual crystal approximation
RA	Random alloy
